# 3-Butyl-2-phenyl-1,3-thia­zolidine-1,4-dione

**DOI:** 10.1107/S1600536810018003

**Published:** 2010-05-22

**Authors:** Qiang Wang, Zhouqin Xu, Yanchun Sun

**Affiliations:** a Department of Physics–Chemistry, Henan Polytechnic University, Jiao Zuo 454000, People’s Republic of China; b Department of Medicine, Hebi College of Vocation and Technology, He Bi 458030, People’s Republic of China

## Abstract

In the title compound, C_13_H_17_NO_2_S, the thia­zolidine-1,4-dione ring adopts an envelope conformation with the S atom lying 0.631 (4) Å out of the plane formed by the other four ring atoms; the phenyl ring is almost perpendicular [88.74 (8)°] with respect to the ring C—C—N—C atoms and the butyl chain is in a fully extended conformation. In the crystal, a supra­molecular two-dimensional arrangement arises from weak inter­molecular C—H⋯O inter­actions.

## Related literature

For related structures, see: Wang *et al.* (2009 ▶); Xu *et al.* (2009 ▶). For synthetic procedures, see: Johnson *et al.* (1983 ▶); Srivastava *et al.* (2002 ▶). 
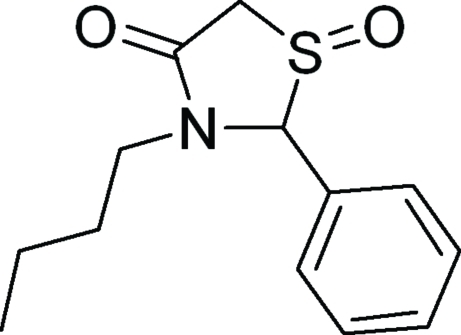


## Experimental

### 

#### Crystal data

C_13_H_17_NO_2_S
*M* *_r_* = 251.34
Monoclinic, 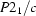

*a* = 13.8335 (5) Å
*b* = 8.7461 (3) Å
*c* = 12.3853 (4) Å
β = 114.773 (2)°
*V* = 1360.59 (8) Å^3^
*Z* = 4
Mo *K*α radiation
μ = 0.23 mm^−1^
*T* = 297 K
0.28 × 0.26 × 0.20 mm

#### Data collection

Bruker APEXII CCD area-detector diffractometer
Absorption correction: multi-scan (*SADABS*; Sheldrick, 1997 ▶) *T*_min_ = 0.939, *T*_max_ = 0.956
15990 measured reflections
3118 independent reflections
2169 reflections with *I* > 2σ(*I*)
*R*_int_ = 0.030

#### Refinement

*R*[*F* ^2^ > 2σ(*F* ^2^)] = 0.050
*wR*(*F* ^2^) = 0.159
*S* = 1.14
3118 reflections
155 parameters
6 restraints
H-atom parameters constrained
Δρ_max_ = 0.34 e Å^−3^
Δρ_min_ = −0.29 e Å^−3^

### 

Data collection: *APEX2* (Bruker, 2003 ▶); cell refinement: *SAINT* (Bruker, 2001 ▶); data reduction: *SAINT*; program(s) used to solve structure: *SHELXS97* (Sheldrick, 2008 ▶); program(s) used to refine structure: *SHELXL97* (Sheldrick, 2008 ▶); molecular graphics: *SHELXTL* (Sheldrick, 2008 ▶); software used to prepare material for publication: *SHELXTL*.

## Supplementary Material

Crystal structure: contains datablocks I, global. DOI: 10.1107/S1600536810018003/pv2278sup1.cif

Structure factors: contains datablocks I. DOI: 10.1107/S1600536810018003/pv2278Isup2.hkl

Additional supplementary materials:  crystallographic information; 3D view; checkCIF report

## Figures and Tables

**Table 1:** Hydrogen-bond geometry (Å, °)

*D*—H⋯*A*	*D*—H	H⋯*A*	*D*⋯*A*	*D*—H⋯*A*
C2—H2*B*⋯O1^i^	0.97	2.43	3.246 (3)	142
C3—H3⋯O2^ii^	0.98	2.34	3.311 (3)	172
C11—H11*B*⋯O2^ii^	0.97	2.59	3.548 (4)	169

Symmetry codes: (i) 
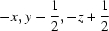
; (ii) 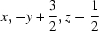.

**Fig. 1.**
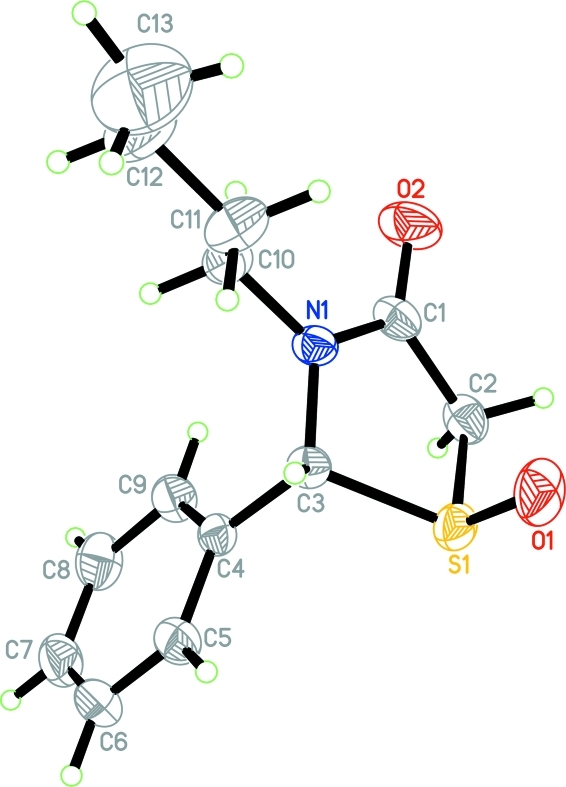
The molecular structure of the title molecule, with the atom-numbering scheme. Displacement ellipsoids are drawn at the 30% probability level.

**Fig. 2.**
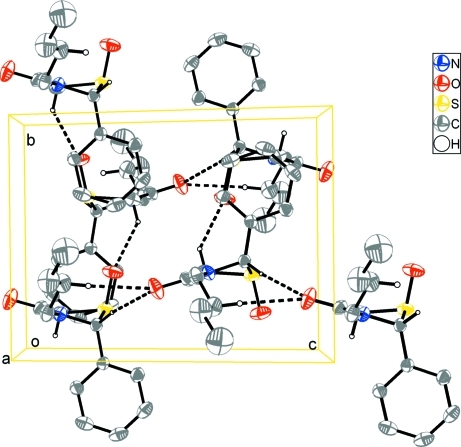
A view of the unit cell packing of the title compound down the a-axis showing hydrogen bonds as dashed lines.

## supplementary crystallographic information

### Comment

As a part of our programme studying the new applications of cyclic sulfoxide derivatives in medicne (Wang, *et al.*, 2009; Xu, *et al.*, 2009), we report herein the crystal structure of the title compounds.

The molecular structure of the title compound is shown in Fig. 1. The thiazolidin-4-one ring adopts an envelope conformation with S lying 0.631 (4) Å out of the plane formed by the rest of the ring atoms; the phenyl ring is oriented at right angles (88.74 (8)°) with respect to the ring (C2/C3/N1/C1) atoms. The butyl chain is in a fully extended conformation. The crystal packing (Fig. 2) consists of a two-dimensional network in the *a-c*-plane generated by intermolecular interactions of the weak C—H···O hydrogen bonds.

### Experimental

All reagents were of analytical grade. The title compound was prepared according to literature methods (Srivastava *et al.*, 2002; Johnson *et al.*, 1983). It was characterized by recording its infrared spectra and elemental analyses. Single crystals of the title compound were obtained by slow evaporation of its chloroform solution at room temperature.

### Refinement

All H atoms bonded to C atoms were calculated in idealized position with C—H = 0.93-0.98 Å and refined in riding mode on their parent atoms with *U*_iso_(H) values of 1.2*U*_eq_(C).

### Crystal data

**Table:**

C_13_H_17_NO_2_S	*F*(000) = 536
*M**_r_* = 251.34	*D*_x_ = 1.227 Mg m^−^^3^
Monoclinic, *P*2_1_/*c*	Mo *K*α radiation, λ = 0.71073 Å
Hall symbol: -P 2ybc	Cell parameters from 5947 reflections
*a* = 13.8335 (5) Å	θ = 2.8–25.9°
*b* = 8.7461 (3) Å	µ = 0.23 mm^−^^1^
*c* = 12.3853 (4) Å	*T* = 297 K
β = 114.773 (2)°	Block, yellow
*V* = 1360.59 (8) Å^3^	0.28 × 0.26 × 0.20 mm
*Z* = 4	

### Data collection

**Table:**

Bruker APEXII CCD area-detector diffractometer	3118 independent reflections
Radiation source: fine-focus sealed tube	2169 reflections with *I* > 2σ(*I*)
graphite	*R*_int_ = 0.030
φ and ω scans	θ_max_ = 27.5°, θ_min_ = 1.6°
Absorption correction: multi-scan (*SADABS*; Sheldrick, 1997)	*h* = −17→12
*T*_min_ = 0.939, *T*_max_ = 0.956	*k* = −11→11
15990 measured reflections	*l* = −15→16

### Refinement

**Table:**

Refinement on *F*^2^	Primary atom site location: structure-invariant direct methods
Least-squares matrix: full	Secondary atom site location: difference Fourier map
*R*[*F*^2^ > 2σ(*F*^2^)] = 0.050	Hydrogen site location: inferred from neighbouring sites
*wR*(*F*^2^) = 0.159	H-atom parameters constrained
*S* = 1.14	*w* = 1/[σ^2^(*F*_o_^2^) + (0.0833*P*)^2^ + 0.1042*P*] where *P* = (*F*_o_^2^ + 2*F*_c_^2^)/3
3118 reflections	(Δ/σ)_max_ < 0.001
155 parameters	Δρ_max_ = 0.34 e Å^−^^3^
6 restraints	Δρ_min_ = −0.28 e Å^−^^3^

### Special details

**Table:**

Geometry. All esds (except the esd in the dihedral angle between two l.s. planes) are estimated using the full covariance matrix. The cell esds are taken into account individually in the estimation of esds in distances, angles and torsion angles; correlations between esds in cell parameters are only used when they are defined by crystal symmetry. An approximate (isotropic) treatment of cell esds is used for estimating esds involving l.s. planes.
Refinement. Refinement of *F*^2^ against ALL reflections. The weighted *R*-factor *wR* and goodness of fit *S* are based on *F*^2^, conventional *R*-factors *R* are based on *F*, with *F* set to zero for negative *F*^2^. The threshold expression of *F*^2^ > σ(*F*^2^) is used only for calculating *R*-factors(gt) *etc*. and is not relevant to the choice of reflections for refinement. *R*-factors based on *F*^2^ are statistically about twice as large as those based on *F*, and *R*– factors based on ALL data will be even larger.

### Fractional atomic coordinates and isotropic or equivalent isotropic displacement parameters (Å^2^)

**Table:**

	*x*	*y*	*z*	*U*_iso_*/*U*_eq_	
N1	0.25227 (13)	0.66161 (18)	0.36775 (14)	0.0437 (4)	
O1	0.04410 (14)	0.84045 (17)	0.19284 (19)	0.0788 (6)	
O2	0.24851 (16)	0.7278 (2)	0.54296 (16)	0.0820 (6)	
S1	0.04960 (4)	0.67497 (6)	0.22013 (5)	0.0522 (2)	
C1	0.20313 (18)	0.6887 (2)	0.4393 (2)	0.0514 (5)	
C2	0.08545 (18)	0.6614 (2)	0.3755 (2)	0.0553 (6)	
H2A	0.0469	0.7371	0.3993	0.066*	
H2B	0.0678	0.5608	0.3949	0.066*	
C3	0.18626 (14)	0.6127 (2)	0.24789 (16)	0.0410 (5)	
H3	0.2085	0.6679	0.1933	0.049*	
C4	0.18891 (14)	0.4445 (2)	0.22558 (16)	0.0393 (4)	
C5	0.15706 (17)	0.3929 (2)	0.10953 (19)	0.0525 (5)	
H5	0.1360	0.4631	0.0474	0.063*	
C6	0.1564 (2)	0.2395 (3)	0.0856 (2)	0.0650 (7)	
H6	0.1346	0.2067	0.0075	0.078*	
C7	0.1875 (2)	0.1350 (3)	0.1756 (3)	0.0692 (7)	
H7	0.1875	0.0313	0.1589	0.083*	
C8	0.2186 (2)	0.1831 (3)	0.2906 (3)	0.0677 (7)	
H8	0.2387	0.1118	0.3518	0.081*	
C9	0.22036 (17)	0.3375 (2)	0.3161 (2)	0.0531 (6)	
H9	0.2428	0.3694	0.3945	0.064*	
C10	0.36729 (17)	0.6710 (3)	0.4092 (2)	0.0577 (6)	
H10A	0.3932	0.5761	0.3906	0.069*	
H10B	0.3996	0.6830	0.4949	0.069*	
C11	0.4012 (2)	0.8007 (3)	0.3546 (3)	0.0734 (7)	
H11A	0.3744	0.8954	0.3726	0.088*	
H11B	0.3689	0.7882	0.2689	0.088*	
C12	0.5200 (2)	0.8135 (4)	0.3968 (3)	0.1025 (11)	
H12A	0.5521	0.8311	0.4820	0.123*	
H12B	0.5473	0.7173	0.3821	0.123*	
C13	0.5527 (3)	0.9381 (6)	0.3380 (4)	0.1532 (17)	
H13A	0.5328	0.9122	0.2561	0.184*	
H13B	0.6284	0.9516	0.3773	0.184*	
H13C	0.5179	1.0314	0.3425	0.184*	

### Atomic displacement parameters (Å^2^)

**Table:**

	*U*^11^	*U*^22^	*U*^33^	*U*^12^	*U*^13^	*U*^23^
N1	0.0431 (9)	0.0507 (10)	0.0389 (9)	−0.0063 (7)	0.0189 (7)	−0.0076 (7)
O1	0.0751 (12)	0.0415 (10)	0.1114 (15)	0.0132 (7)	0.0307 (11)	0.0173 (9)
O2	0.0877 (14)	0.1101 (16)	0.0556 (11)	−0.0193 (11)	0.0372 (10)	−0.0332 (10)
S1	0.0453 (3)	0.0400 (3)	0.0641 (4)	0.0035 (2)	0.0156 (3)	0.0018 (2)
C1	0.0614 (14)	0.0500 (12)	0.0506 (14)	−0.0080 (10)	0.0312 (11)	−0.0118 (10)
C2	0.0566 (13)	0.0484 (12)	0.0733 (16)	−0.0076 (9)	0.0395 (12)	−0.0104 (10)
C3	0.0437 (10)	0.0447 (10)	0.0367 (11)	−0.0019 (8)	0.0190 (9)	0.0008 (8)
C4	0.0381 (10)	0.0426 (10)	0.0403 (11)	0.0019 (8)	0.0195 (8)	−0.0010 (8)
C5	0.0572 (13)	0.0567 (13)	0.0464 (13)	0.0022 (10)	0.0243 (10)	−0.0042 (10)
C6	0.0661 (16)	0.0669 (16)	0.0636 (16)	−0.0004 (12)	0.0288 (13)	−0.0227 (13)
C7	0.0654 (15)	0.0489 (13)	0.094 (2)	0.0026 (11)	0.0339 (15)	−0.0165 (14)
C8	0.0723 (17)	0.0492 (14)	0.083 (2)	0.0130 (11)	0.0336 (14)	0.0151 (12)
C9	0.0617 (14)	0.0517 (12)	0.0472 (13)	0.0068 (10)	0.0241 (11)	0.0037 (9)
C10	0.0464 (12)	0.0706 (15)	0.0527 (14)	−0.0020 (10)	0.0174 (10)	−0.0063 (11)
C11	0.0514 (15)	0.0891 (18)	0.0734 (18)	−0.0103 (13)	0.0200 (13)	0.0048 (14)
C12	0.0567 (17)	0.137 (3)	0.113 (3)	−0.0209 (17)	0.0347 (18)	−0.005 (2)
C13	0.127 (2)	0.179 (3)	0.168 (3)	−0.041 (2)	0.076 (2)	0.009 (2)

### Geometric parameters (Å, °)

**Table:**

N1—C1	1.345 (2)	C7—C8	1.370 (4)
N1—C3	1.444 (2)	C7—H7	0.9300
N1—C10	1.455 (3)	C8—C9	1.385 (3)
O1—S1	1.4809 (16)	C8—H8	0.9300
O2—C1	1.218 (3)	C9—H9	0.9300
S1—C2	1.779 (2)	C10—C11	1.493 (3)
S1—C3	1.8559 (18)	C10—H10A	0.9700
C1—C2	1.501 (3)	C10—H10B	0.9700
C2—H2A	0.9700	C11—C12	1.507 (4)
C2—H2B	0.9700	C11—H11A	0.9700
C3—C4	1.500 (3)	C11—H11B	0.9700
C3—H3	0.9800	C12—C13	1.482 (5)
C4—C9	1.383 (3)	C12—H12A	0.9700
C4—C5	1.390 (3)	C12—H12B	0.9700
C5—C6	1.374 (3)	C13—H13A	0.9600
C5—H5	0.9300	C13—H13B	0.9600
C6—C7	1.364 (4)	C13—H13C	0.9600
C6—H6	0.9300		
			
C1—N1—C3	116.95 (17)	C8—C7—H7	120.1
C1—N1—C10	122.52 (18)	C7—C8—C9	120.3 (2)
C3—N1—C10	120.42 (15)	C7—C8—H8	119.8
O1—S1—C2	106.05 (11)	C9—C8—H8	119.8
O1—S1—C3	106.27 (10)	C8—C9—C4	120.3 (2)
C2—S1—C3	88.82 (9)	C8—C9—H9	119.8
O2—C1—N1	124.4 (2)	C4—C9—H9	119.8
O2—C1—C2	124.46 (19)	N1—C10—C11	112.81 (18)
N1—C1—C2	111.17 (19)	N1—C10—H10A	109.0
C1—C2—S1	107.95 (14)	C11—C10—H10A	109.0
C1—C2—H2A	110.1	N1—C10—H10B	109.0
S1—C2—H2A	110.1	C11—C10—H10B	109.0
C1—C2—H2B	110.1	H10A—C10—H10B	107.8
S1—C2—H2B	110.1	C10—C11—C12	113.8 (2)
H2A—C2—H2B	108.4	C10—C11—H11A	108.8
N1—C3—C4	115.24 (16)	C12—C11—H11A	108.8
N1—C3—S1	105.05 (12)	C10—C11—H11B	108.8
C4—C3—S1	110.82 (12)	C12—C11—H11B	108.8
N1—C3—H3	108.5	H11A—C11—H11B	107.7
C4—C3—H3	108.5	C13—C12—C11	113.4 (3)
S1—C3—H3	108.5	C13—C12—H12A	108.9
C9—C4—C5	118.34 (19)	C11—C12—H12A	108.9
C9—C4—C3	122.52 (18)	C13—C12—H12B	108.9
C5—C4—C3	119.13 (17)	C11—C12—H12B	108.9
C6—C5—C4	120.7 (2)	H12A—C12—H12B	107.7
C6—C5—H5	119.6	C12—C13—H13A	109.5
C4—C5—H5	119.6	C12—C13—H13B	109.5
C7—C6—C5	120.5 (2)	H13A—C13—H13B	109.5
C7—C6—H6	119.8	C12—C13—H13C	109.5
C5—C6—H6	119.8	H13A—C13—H13C	109.5
C6—C7—C8	119.8 (2)	H13B—C13—H13C	109.5
C6—C7—H7	120.1		
			
C3—N1—C1—O2	178.6 (2)	N1—C3—C4—C9	21.3 (3)
C10—N1—C1—O2	2.4 (3)	S1—C3—C4—C9	−97.9 (2)
C3—N1—C1—C2	−0.3 (2)	N1—C3—C4—C5	−159.80 (16)
C10—N1—C1—C2	−176.47 (18)	S1—C3—C4—C5	81.10 (18)
O2—C1—C2—S1	159.4 (2)	C9—C4—C5—C6	0.3 (3)
N1—C1—C2—S1	−21.8 (2)	C3—C4—C5—C6	−178.67 (18)
O1—S1—C2—C1	−78.65 (16)	C4—C5—C6—C7	−0.2 (3)
C3—S1—C2—C1	27.94 (15)	C5—C6—C7—C8	0.6 (4)
C1—N1—C3—C4	−101.4 (2)	C6—C7—C8—C9	−1.0 (4)
C10—N1—C3—C4	74.9 (2)	C7—C8—C9—C4	1.1 (3)
C1—N1—C3—S1	20.9 (2)	C5—C4—C9—C8	−0.7 (3)
C10—N1—C3—S1	−162.82 (15)	C3—C4—C9—C8	178.21 (18)
O1—S1—C3—N1	78.93 (14)	C1—N1—C10—C11	−112.5 (2)
C2—S1—C3—N1	−27.45 (13)	C3—N1—C10—C11	71.4 (2)
O1—S1—C3—C4	−155.99 (14)	N1—C10—C11—C12	179.6 (2)
C2—S1—C3—C4	97.63 (14)	C10—C11—C12—C13	177.3 (3)

### Hydrogen-bond geometry (Å, °)

**Table:**

*D*—H···*A*	*D*—H	H···*A*	*D*···*A*	*D*—H···*A*
C2—H2B···O1^i^	0.97	2.43	3.246 (3)	142
C3—H3···O2^ii^	0.98	2.34	3.311 (3)	172
C9—H9···N1	0.93	2.59	2.899 (2)	100
C10—H10B···O2	0.97	2.43	2.824 (3)	104
C11—H11B···O2^ii^	0.97	2.59	3.548 (4)	169

Symmetry codes: (i) −*x*, *y*−1/2, −*z*+1/2; (ii) *x*, −*y*+3/2, *z*−1/2.
